# Effect of Moderate-to-High-Intensity Interval Aerobic Exercise on Clinical Symptoms During the Menstrual Cycle: A Pilot Randomized Controlled Trial

**DOI:** 10.3390/healthcare13233113

**Published:** 2025-11-30

**Authors:** Alejandra Pio-Soria, Doaa Zahran, Alberto Roldán-Ruiz

**Affiliations:** 1 Departamento de Fisioterapia, Facultad de Ciencias de la Salud, Universidad Francisco de Vitoria, Ctra. Pozuelo-Majadahonda Km 1,800, Pozuelo de Alarcón, 28223 Madrid, Spain; alex.pio.soria@gmail.com; 2 Institute of Health and Sport Sciences, Faculty of Health Science, Universidad Francisco de Vitoria, 28223 Madrid, Spain

**Keywords:** primary dysmenorrhea, aerobic exercise, menstrual pain, quality of life, sleep quality, stress

## Abstract

Introduction: Primary dysmenorrhea is one of the most prevalent gynecological disorders. It has been shown to negatively impact quality of life and overall wellbeing, as recent studies have associated stress and physical inactivity with both the onset and severity of menstrual pain. Objective: To analyze the effectiveness of a moderate-to-high-intensity interval aerobic exercise on menstrual pain intensity, menstrual-related quality of life, general health-related quality of life, sleep quality, stress and anxiety, and drug intake in young women with primary dysmenorrhea. Methodology: A total of 20 women were randomly allocated to either the exercise (N = 10) or control group (N = 10). Primary outcomes were menstrual pain intensity and menstrual-related quality of life. Secondary outcomes included general health-related quality of life, sleep quality, stress and anxiety, and drug intake. A supervised moderate-to-high-intensity interval aerobic exercise program on a stationary bicycle was carried out twice a week, for eight weeks, in young women with regular cycles and no diagnosed medical conditions. Patients were assessed at baseline, and at 1- and 2-month follow-ups. Results: At 8 weeks, 17 women completed the follow-up. Analyses showed statistically significant differences in favor of moderate-to-high-intensity interval aerobic exercise at 2-month follow-up for menstrual pain intensity, with a large size effect. Significant changes were also found in menstrual-related quality of life at both follow-ups in the exercise group, but they were not superior to the control group. No significant changes were observed for the rest of the variables in either group. Conclusions: The results from this pilot study suggest that the moderate-to-high-intensity interval aerobic exercise program is effective in reducing menstrual pain intensity at 2-month follow-ups. Future randomized controlled trials with larger samples are required to confirm the results.

## 1. Introduction

Primary dysmenorrhea (PD) is one of the most common gynecological disorders in women and usually begins within the first years after menarche [1,2]. It is characterized by recurrent, colicky, sharp, and spasmodic pain located in the pelvic area, radiating to the lumbosacral region and/or both thighs [3,4,5]. Additionally, it may be associated with headaches, nausea, vomiting, diarrhea, fatigue, and myalgia, and its occurrence is often correlated with early menarche, longer and heavier menstrual flow, stress, depression, anxiety, smoking, higher body mass index (BMI), sedentary lifestyle, premenstrual syndrome, and family history of dysmenorrhea [6,7].

Menstrual pain usually begins just before or within the first few hours of menstruation and generally lasts up to 72 h, peaking in intensity between 24 and 48 h after menstruation onset [3]. It is estimated that between 45% and 95% of women worldwide are affected by this condition [5]. In Spain, the prevalence is approximately 56% to 62% in the general population and reaches up to 75% among university-aged women [8].

The etiology of PD remains incompletely understood [5]. However, the most widely accepted hypothesis implicates elevated prostaglandin production in the endometrium during menstruation [2]. Specifically, a decline in progesterone levels at the end of the luteal phase has been associated with increased synthesis of prostaglandins PGF2α and PGE2, which induce uterine vasoconstriction and myometrial contractions, while simultaneous leukocyte infiltration and proinflammatory cytokines contribute to the inflammatory milieu characteristic of PD [4]. Alternative theories propose that dysregulation of central pain processing mechanisms may also play a role, resulting in heightened pain perception across different phases of the menstrual cycle, independent of peripheral nociceptive input [2].

Various studies have shown that menstrual pain negatively affects women’s quality of life compared to those without PD, causing work and school absenteeism, difficulty concentrating, and poor academic performance [6]. However, most women do not seek treatment either because they consider these symptoms normal, opt for self-medication with analgesics, anti-inflammatories, or home remedies, are unaware of treatment options, or because they feel fear or embarrassment about seeking medical care; reasons that might underscore why the prevalence of this disorder may even be underestimated [6,9].

The primary treatment for PD is based on inhibiting prostaglandins through the use of nonsteroidal anti-inflammatory drugs [4,10]. However, continued use of these can lead to issues such as gastric mucosal damage, kidney or liver failure, NSAID hypersensitivity, and other adverse effects like nausea, headache, or dizziness [2]. The second-line treatment involves the use of hormonal contraceptives which, by inhibiting ovulation, reduce progesterone production and consequently the rise in prostaglandins. However, up to 30.8% of women experience side effects such as weight gain, headaches, irregular spotting, nausea, and/or alopecia, and contraceptives have also been associated with increased breast cancer risk [5,11,12].

Recent studies have shown that aerobic exercise can help relieve menstrual pain through various physiological mechanisms, for example, by increasing progesterone and decreasing prostaglandins and proinflammatory cytokines [13]. This would reduce the inflammatory environment and, consequently, the severity of symptoms, possibly through the activation of the endocannabinoid system and the release of endorphins, which lower pain perception [14]. Additionally, aerobic exercise not only reduces levels of stress and anxiety and improves quality of life—factors associated with PD severity—but is also more effective than other exercise modalities such as yoga or stretching in reducing pain and the use of analgesics [15].

Therefore, the objective of the present study was to analyze the effectiveness of an moderate-to-high-intensity interval aerobic exercise on menstrual pain intensity, menstrual-related quality of life, general health-related quality of life, sleep quality, stress and anxiety, and drug intake in young women with primary dysmenorrhea.

## 2. Methods

### 2.1. Design

A single-blind pilot study of a randomized clinical trial was conducted. This research was prospectively registered on 27 January 2025 on www.clinicaltrials.gov (NCT06804473) and approved by the Research Ethics Committee of the Francisco de Vitoria University, Madrid, Spain (9/2025).

### 2.2. Participants

Consecutive women with a clinical diagnosis of PD were screened for eligibility criteria. The diagnosis was confirmed based on data obtained from the patient’s medical history: personal history, onset of pain associated with menstruation, pain characteristics, concomitant symptoms, and the absence of signs typically associated with secondary dysmenorrhea, such as irregular bleeding or pain that does not improve with NSAIDs.

To be eligible, individuals had to meet the following criteria: aged between 18 and 26 years, <3 h/week of moderate-intensity physical activity, self-reported regular menstrual cycle (24–38 days), and pain score ≥ 4 on the Visual Analogue Scale (VAS). On the other hand, participants were excluded if any of the following were noted: use of contraceptive pills in the past 3 months; presence of an intra-uterine device; pregnancy or breastfeeding in the past 3 months; amenorrhea; smoking; regular alcohol consumption; chronic endocrine, digestive, neurological, or cardiovascular diseases; and/or psychiatric disorders, as well as any other medical diagnosis that contraindicates physical activity.

Participants who met the selection criteria signed an informed consent and completed an initial questionnaire designed to collect data on demographic characteristics, medical history, details about their menstrual cycle, and their level of physical activity. Furthermore, the study was conducted in accordance with the principles of the Declaration of Helsinki.

### 2.3. Treatment Allocation

Participants were randomly assigned to either the experimental group (moderate-to-high-intensity interval aerobic exercise) or the control group (no intervention). Allocation concealment was ensured using a computer-generated randomization list created prior to participant recruitment by a researcher not involved in enrollment. Individual, sequentially numbered cards with group assignments were prepared, folded, and placed in sealed opaque envelopes. Another researcher, blinded to participant recruitment, opened the envelopes and assigned the participants to their respective groups. The researchers responsible for collecting follow-up data were blinded to group allocation. Due to the nature of the intervention and self-reported outcomes, it was not possible to blind the participants or all of the researchers. However, the person in charge of statistical analysis was also blinded to the participants’ group assignments.

### 2.4. Interventions

Patients allocated to the control group received no treatment (wait and see). On the other hand, participants in the experimental group performed a moderate-to-high-intensity interval aerobic exercise on a stationary bicycle with adjustable resistance at the Therapeutic Exercise Laboratory of the Department of Physiotherapy, Universidad Francisco de Vitoria, Madrid, Spain. All exercise sessions were carried out between 15:00 and 17:00 to minimize circadian variability in physiological responses such as heart rate, perceived exertion, and fatigue. This timing was kept consistent across all participants throughout the intervention period. Menstrual cycle phases were not tracked for scheduling exercise sessions. All participants performed the same protocol regardless of cycle phase, prioritizing feasibility and adherence in this pilot design.

The moderate-to-high-intensity interval aerobic exercise consisted of 60–75% of maximum heart rate (HRmax) training, for 26 min per session, twice a week. Participants carried out 4 intervals, each composed of two phases: an active phase (5 min) at 60–75% HRmax, and an active recovery phase (1 min and 30 s) at 30–50% HRmax. Before starting, participants completed a 5-min warm-up on the stationary bicycle with a gradual increase in HRmax: 3 min at 50–60%, followed by 2 min at 60–70% HRmax. At the end of the session, a 5-min cool-down was performed, progressively reducing intensity to 30–50% HRmax. Sessions were only conducted when participants’ perceived pain was less than 8 on the Visual Analogue Scale (VAS).

Exercise intensity was individualized for each participant by calculating their HRmax using the following formula: HRmax = 226—age [16]. Heart rate was monitored throughout the sessions using an Apple Watch Series 6, Model A2292 (Apple Inc., Cupertino, CA, USA), to ensure participants exercised within the prescribed intensity range. Additionally, the modified Borg scale, ranging from 0 (no exertion) to 10 (maximum exertion), was used to assess perceived exertion as a complementary method to HRmax monitoring [17]. Subjective perceived exertion during the active phase was maintained within the 4–7 range on the Borg scale, corresponding to moderate-to-high self-reported perceived intensity. Interval timing was precisely managed using the smartphone application “Tabata Timer” to ensure proper execution. No specific session adjustments were made based on the menstrual cycle phase; participants performed the same exercise with consistent parameters throughout the entire cycle. If heart rate or perceived exertion exceeded the target thresholds, the pedaling intensity or bike resistance was reduced to align with the target values.

### 2.5. Outcomes

Independent variables collected included age, weight, height, age at menarche, number of days of bleeding, and the presence or absence of premenstrual symptoms. With regard to the dependent variables, three assessments were conducted: pre-intervention, at 4-weeks follow-up, and at 8-weeks follow-up.

#### 2.5.1. Primary Variables

Menstrual pain intensity was assessed using the VAS, which includes a rating from 0 to 10, where a higher score indicates greater perceived pain intensity. The VAS has demonstrated excellent test–retest reliability (r = 0.94) and high convergent validity (r = 0.62–0.91) [18].

Menstrual-related quality of life was assessed using the Menstrual Quality of Life Questionnaire (CVM–22), which evaluates perceived health, physical and functional well-being, psychological and cognitive well-being, and menstrual symptoms. A higher score indicates worse quality of life during menstruation. The CVM–22 has shown a reliability of 0.90 and a total internal consistency of 0.917 in Spanish-speaking women aged 18 to 35 [19] (Annex 4).

#### 2.5.2. Secondary Variables

Quality of life was measured using the Short Form Health Survey (SF-12), which assesses physical and mental components through 12 items. Higher scores indicate poorer perceived quality of life. The SF-12 has demonstrated test–retest reliability in the physical component (r = 0.89) and in the mental component (r = 0.76), as well as construct validity through correlations with the SF-36 (R^2^ = 0.904–0.938) [20,21].

Sleep quality was measured using the Women’s Health Initiative Insomnia Rating Scale (WHIIRS), which evaluates the severity of insomnia through 5 items rated from 0 (never) to 4 (always). A higher score indicates moderate-to-severe insomnia. The WHIIRS has demonstrated high test–retest reliability (r = 0.96) [22].

Stress and anxiety were assessed using the Depression, Anxiety, and Stress Scale–21 (DASS–21), a 21-item self-report scale with responses ranging from 0 to 3. Higher scores indicate greater psychological distress. The DASS–21 has shown good internal consistency (α = 0.73–0.81) and construct validity in university populations [23].

Drug intake was recorded, determining the quantity of pain medication consumed during the evaluated menstrual cycles.

Exercise adherence was measured by tracking attendance at the pre-scheduled exercise sessions.

### 2.6. Sample Size

The sample size was previously calculated through the G-Power program, assuming an effect size of 0.35 for the primary outcome, a statistical power of 80% and an alpha error probability of 0.05. This showed that at least 44 participants were needed for the whole sample [24]. However, as this was a pilot study, we were unable to recruit the full calculated sample size. The primary aim was to obtain the highest possible number of participants within the recruitment period to assess feasibility and preliminary efficacy, providing essential data to inform the design and sample size estimation of future randomized controlled trials meeting sample size requirements.

### 2.7. Statistical Analysis

Statistical analysis was performed using SPSS software, version 28.0 (Chicago, IL, USA). Quantitative baseline sociodemographic and clinical variables were described using Means and Standard Deviations when normally distributed, or Medians and Interquartile Ranges otherwise. Qualitative variables were presented as absolute and relative frequencies. Comparisons between groups were conducted using independent samples Student’s t-tests or Mann–Whitney U tests, as appropriate. For categorical variables, chi-square tests or Fisher’s exact tests were employed.

Normality assumptions for the dependent variables were assessed using the Shapiro–Wilk test. Inferential analysis involved repeated-measures analysis of variance (ANOVA) for normally distributed variables and the Friedman test for non-normally distributed variables. Results were estimated with a 95% confidence interval, considering a *p*-value < 0.05 as statistically significant. Effect size was measured using partial eta squared (η^2^), with reference values of 0.01 (small), 0.06 (moderate), and ≥0.14 (large). Bivariate analyses of associations between baseline characteristics and changes in dependent variables were determined using Pearson correlation coefficients.

## 3. Results

**Figure 1:**
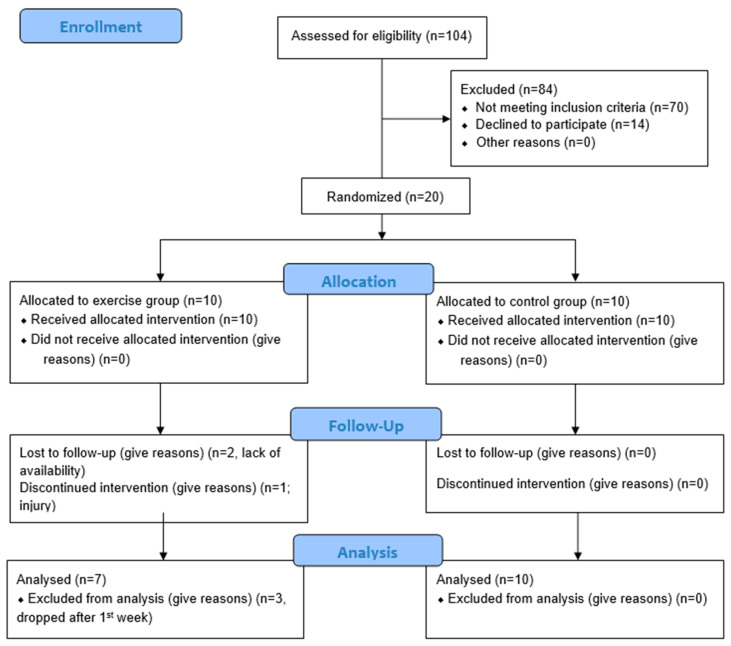
CONSORT flow diagram.

A total of 104 young women volunteered to participate in the study, of whom 70 were excluded for not meeting the inclusion criteria, and 14 finally declined to participate (Figure 1). The final eligible sample consisted of 20 subjects, of which, after randomization and just prior to the start of intervention, 2 participants withdrew due to scheduling conflicts and 1 due to an injury unrelated to the study, leaving 7 women in the experimental group and 10 in the control group. Thus, between 8 September and 3 November 2025, 17 women completed the study. The experimental group showed 100% adherence to the moderate-to-high-intensity interval aerobic exercise program, completing all scheduled sessions.

The sociodemographic and baseline clinical characteristics of the sample are shown in Table 1. No significant differences were observed between groups in any of the baseline characteristics (*p* > 0.05), indicating that groups were comparable prior to the intervention.

With respect to inferential analyses, all pre–post changes in data in both groups can be found in Table 2.

### 3.1. Menstrual Pain Intensity

After completing the first 4 weeks of training, the experimental group showed a statistically significant reduction in pain intensity with a moderate effect size (*p* < 0.001; n^2^p = 0.09), whereas the control group showed no significant changes (*p* = 0.62). However, no statistically significant differences were observed in the pre–post comparison between groups (*p* = 0.24) at this follow-up.

**Figure 2:**
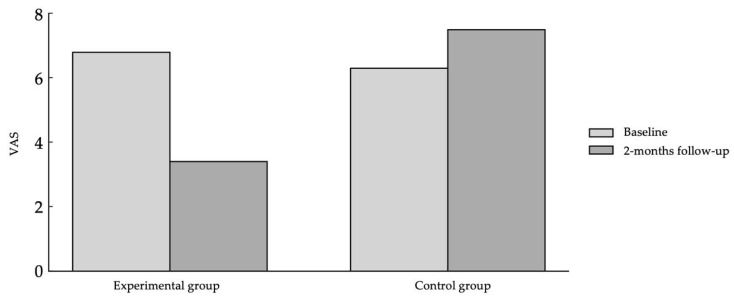
Pre–post menstrual pain intensity changes after intervention.

At the 8-week follow-up, the experimental group also demonstrated a statistically significant reduction in pain intensity with a large effect size (*p* < 0.001; n^2^p = 0.89), while the control group continued to show no significant changes (*p* = 0.35). In this case, statistically significant differences were found in the pre–post comparison between groups (*p* = 0.001), with a large effect size (η^2^p = 0.36). Pre–post means in both groups can be found in Figure 2.

### 3.2. Menstruation-Related Quality of Life

Both at 4-week and 8-week follow-ups, the experimental group showed statistically significant changes with a large effect size (*p* < 0.001; n^2^p = 0.70), whereas the control group did not show significant changes (*p* = 0.40; *p* = 0.06). However, no statistically significant differences were found in the pre–post comparison between groups in any case (*p* = 0.98; *p* = 0.38).

### 3.3. General Quality of Life

At both 4- and 8-week follow-ups, no statistically significant changes were observed in the physical dimension of quality of life for the experimental group (*p* > 0.05) or the control group (*p* = 0.67; *p* > 0.05), and no significant differences were found between the groups (*p* = 0.31; *p* = 0.12). Similarly, in the mental dimension, neither group showed significant changes (*p* > 0.05), and there were no differences between groups at both follow-ups (*p* = 0.58; *p* = 0.50).

### 3.4. Sleep Quality

After 4 weeks of intervention, neither the experimental group nor the control group showed statistically significant changes (*p* > 0.05), nor were there any significant differences between groups (*p* = 0.94). At 8 weeks, no significant changes were observed in either group (*p* > 0.05) and no statistically significant differences were found between groups (*p* = 0.78).

### 3.5. Anxiety and Stress

At both 4- and 8-week follow-ups, no statistically significant changes were observed in stress levels for the experimental group (*p* > 0.05) or the control group (*p* = 0.59; *p* > 0.05), with no significant differences between groups (*p* = 0.38; *p* = 0.88). Similarly, anxiety levels did not show significant changes in either group at both follow-ups (*p* > 0.05), and no significant between-group differences were found (*p* = 0.78; *p* = 0.53).

### 3.6. Drug Intake

After 4 weeks of intervention, the experimental group did not show statistically significant changes (*p* = 0.24), nor did the control group (*p* > 0.05), and no statistically significant differences were observed between groups (*p* = 0.22). After 8 weeks of intervention, the experimental group did not show statistically significant changes (*p* > 0.05), nor did the control group (*p* = 0.92); again, no statistically significant differences between groups were found (*p* = 0.13).

## 4. Discussion

This is the first randomized clinical trial to evaluate the effect of moderate-to-high-intensity interval aerobic exercise throughout the entire menstrual cycle over two consecutive cycles in young women with PD.

### 4.1. Menstrual Pain Intensity

The findings of this study showed statistically significant differences in menstrual pain intensity when comparing the experimental group to the control group. Improvements exceeded the minimum detectable change established for the VAS, suggesting that moderate-to-high-intensity aerobic exercise was effective in reducing menstrual pain in young women with PD. These results align with several previous studies that implemented similar exercise programs 2–3 times per week for 20 to 30 min over 8 to 12 weeks [15,25,26].

Additionally, Israel et al. (2015) [27] reported greater improvements when exercise was carried out specifically during menstruation days, reinforcing that exercise on days with more pronounced symptoms may also be an indication rather than a contraindication. This analgesic effect may be explained by activation of descending pain inhibitory systems and the release of endogenous opioids, such as serotonin or endocannabinoids, which can inhibit nociceptive transmission at the spinal cord level [28]. Furthermore, regular physical activity reduces pro-inflammatory cytokines (IL-6 and TNF-α) and increases anti-inflammatory cytokines (IL-10), thereby potentially mitigating peripheral and central sensitization [29]. Indeed, hypoalgesic effects have been associated with exercise performed at moderate-to-high intensity for over 10 min [30].

Moreover, Sutar et al. (2016) [31] suggested that activities such as cycling relax the abdominal muscles and improve pelvic blood flow, reducing pressure on pelvic and digestive organs and thereby proposing another hypothesis that might justify menstrual pain reductions.

To the authors’ knowledge, there is only one study that found no positive effects of aerobic exercise in PD patients, in a large sample of 654 university students [32]. This discrepancy may be due to uncontrolled variables such as age, childbirth history, smoking, emotional status, or contraceptive use—factors that have been controlled in the present study.

### 4.2. Menstrual-Related Quality of Life

PD negatively impacts women’s quality of life, particularly during menstruation, and is associated with reduced academic performance in young women [6,33,34].

In our study, no statistically significant differences between groups were observed for this variable. Other studies, however, have found improvements in menstrual-related quality of life, possibly due to differences in exercise modalities (e.g., relaxation-based approaches) or measurement instruments [35,36]. One possible explanation regarding the differences in reported results is that baseline scores were relatively high in our pilot study, suggesting minimal baseline impairment of menstrual-related quality of life and reducing the likelihood of detecting a meaningful improvement in this variable.

### 4.3. General Quality of Life

Previous research has shown that PD can adversely affect perceived overall health, particularly physical functioning, general health perceptions, and pain [37,38].

In our study, no significant between-group differences were observed in either physical or mental dimensions of quality of life. Nonetheless, other studies have reported improvements following longer-duration programs when implementing multimodal interventions combining aerobic exercise with other treatment approaches [25,35,39].

### 4.4. Sleep Quality

Recent evidence indicates a bidirectional relationship between menstrual pain and sleep: poor sleep contributes to fatigue and intensifies pain, as women with insomnia report greater menstrual pain and interference with daily activities [2,40,41].

However, no significant differences between groups were observed in our study for this variable. These findings are consistent with Kannan et al. (2019) [25], who attributed their null results to participants not presenting insomnia at baseline, which is something that also occurred in this research. In contrast, other studies did report improvements in sleep quality after exercise programs, which indicates that conclusions should be drawn cautiously regarding the effects of exercise on sleep quality [42].

### 4.5. Stress and Anxiety

Emotional status is an important factor in managing PD, as anxiety and depression can amplify menstrual symptoms [43,44]. This occurs through the activation of the corticotropin-releasing hormone–adrenocorticotropic hormone–cortisol axis which interferes with follicle-stimulating hormone and luteinizing hormone secretion, reducing progesterone and increasing prostaglandin and estrogen levels, thereby intensifying uterine contractions [45].

In this regard, no significant between-group differences were observed in stress or anxiety levels in our study. These results are consistent with Azima et al. (2015) [46], but contradict the ones obtained by Singh et al. (2023) [47], who found that physical exercise significantly reduces depression, anxiety, and stress symptoms. Similarly, Elbandrawy et al. (2021) [48] observed improvements in emotional symptoms following an exercise program. Differences in study results may be due to variations in sample sizes, with larger samples providing more robust data, as well as differences in the comprehensiveness and frequency of multimodal exercise interventions.

These results may be also influenced by contextual and physiological factors. The intervention coincided with a period of high academic demand, and participants frequently reported stressors such as exams and assignment deadlines during supervised sessions. Academic stress is known to activate the hypothalamic–pituitary–adrenal axis, increasing cortisol and amplifying menstrual symptoms, which could have attenuated potential benefits of exercise. Additionally, hormonal fluctuations across menstrual phases modulate emotional regulation through changes in estrogen and progesterone, affecting mood and stress responses. Altogether, these factors may help explain the absence of significant effects on emotional outcomes in this pilot trial.

### 4.6. Drug Intake

Although drug intake is rarely considered as a variable for analysis in this population [25] and sometimes is even used as an exclusion criteria [35], we believe it should be examined as a relevant clinical outcome that affects patients’ quality of life. Nonetheless, potentially associated with sample size or follow-up limitations of the present study, no significant differences were observed in this variable.

### 4.7. Limitations and Clinical Implications

This pilot randomized clinical trial has several limitations. The short intervention period (8 weeks) and small sample size reduced statistical power, potentially limiting the detection of significant effects on secondary outcomes such as sleep quality, emotional well-being, and quality of life. Additionally, the relatively high baseline scores in these variables may have introduced ceiling effects. Contextual factors such as academic stress, dietary patterns, and other lifestyle factors could have further influenced outcome measures. Although participants reported no additional exercise during supervised sessions, the absence of objective monitoring limits certainty. Similarly, the absence of menstrual phase tracking may limit interpretation of physiological mechanisms, as hormonal fluctuations could modulate pain and exercise responses. Future studies should incorporate phase-specific analyses, standardized activity tracking, and dietary assessment to improve control of potential confounders.

Despite these constraints, the intervention demonstrated a significant reduction in menstrual pain and achieved full adherence, highlighting its feasibility and potential clinical utility as a non-pharmacological strategy for managing primary dysmenorrhea in women. These findings support the inclusion of moderate-to-high-intensity aerobic interval training in menstrual health programs. Larger, long-term trials are needed to validate these results.

## 5. Conclusions

Moderate-to-high-intensity interval aerobic exercise is effective in reducing menstrual pain after 8 weeks of training in young women with primary dysmenorrhea. However, the intervention did not demonstrate significant improvements in menstrual-related quality of life, general quality of life, sleep quality, anxiety, stress, or drug intake. Future studies with larger samples and longer follow-up periods are warranted to confirm and expand upon these findings.

## Figures and Tables

**Table 1:** Sample sociodemographic and baseline clinical characteristics, *n* = 17.

Variable—M (SD)	Data
Age	20.4 (1.2)
Weight (kg)	56.9 (5.4)
Height (cm)	166 (4.8)
Body mass index	20.7 (1.8)
Age at Menarche (years old)	12.3 (1.3)
Working activity, yes/no—*n* (%)	8 (47.1)/9 (52.9)
Days of menstrual bleeding	5.9 (1.1)
Pre-menstrual symptoms, yes/no—*n* (%)	16 (94.1)/1 (5.9)
Drug use, yes/no—*n* (%)	13 (76.5)/4 (23.5)
Pain intensity (VAS)	6.76 (1.4)
Quality of life, physical dimension (SF-12)	51.4 (6.9)
Quality of life, mental dimension (SF-12)	42.6 (11.5)
Anxiety (DASS-21), ME (IQR)	2 (5)
Stress (DASS-21)	7.6 (4.3)
Sleep quality (WHIIRS)	6 (4.1)
Menstrual-related quality of life (CVM-22)	30.7 (15.1)

Abbreviations: M—Mean; SD—Standard Deviation; ME—Median; IQR—Interquartile Range; VAS—Visual Analogue Scale; SF-12—Short Form-12 Health Survey; DASS-21—Depression, Anxiety and Stress Scale; WHIIRS—Women’s Health Initiative Insomnia Rating Scale; CVM-22—Menstrual-Related Quality of Life Questionnaire.

**Table 2:** Within-group changes and between-group differences in outcomes measures at 1-month and 2-month follow-ups.

	Baseline, M (SD)	1-Month Follow-Up					2-Month Follow-Up				
		Within Group		Between Groups			Within Group		Between Groups		
		M (SD)	*p* Value	MD (SD)	*p* Value	η^2^p	M (SD)	*p* Value	M (SD)	*p* Value	η^2^p
Menstrual pain intensity, VAS											
EG	7.0 (1.3)	4.0 (1.5)	**<0.001**	2.5 (1.9)	0.24	0.09	3.3 (1.5)	**<0.001**	3.4 (2.2)	**0.001**	0.36
CG	6.6 (1.5)	6.1 (1.5)	0.62				7.3 (1.12)	0.35			
Menstrual-related quality of life, CVM-22											
EG	35.0 (10.2)	23.8 (9.9)	**<0.001**	15.3 (10.1)	0.98	0.00	14.8 (10.9)	**<0.001**	27.9 (16.7)	0.38	0.05
CG	27.6 (17.6)	30.7 (18.2)	0.40				35.3 (18.1)	0.06			
Quality of life—health dimension, SF-12											
EG	53.5 (8.85)	55.7 (5.0)	1.00	2.3 (7.2)	0.31	0.07	56.0 (2.8)	1.00	0 (2.2)	0.12	0.15
CG	50.0 (5.26)	54.5 (5.9)	0.67				52.5 (5.8)	1.00			
Quality of life—mental dimension, SF-12											
EG	39.7 (11.9)	40.6 (9.8)	1.00	−4.1 (2.2)	0.58	0.02	39.0 (11.3)	1.00	−4.8 (1.2)	0.50	0.03
CG	44.5 (11.4)	41.3 (12.2)	1.00				41.7 (12.0)	1.00			
Sleep quality, WHIIRS											
EG	6.9 (3.08)	5.6 (3.6)	1.00	2.6 (6.6)	0.94	0.00	6.6 (4.4)	1.00	1.5 (8.7)	0.78	0.005
CG	5.4 (4.67)	6.7 (5.3)	1.00				6.9 (3.4)	1.00			
Stress, DASS-21											
EG	7.6 (5.19)	6.3 (5.4)	1.00	4 (4.8)	0.38	0.05	8.4 (6.7)	1.00	0.6 (3.7)	0.88	0.002
CG	7.6 (3.89)	10.3 (6.0)	0.59				9.0 (5.1)	1.00			
Drug intake											
EG	2.6 (2.4)	0.9 (1.5)	0.24	2.4 (4.1)	0.22	0.10	0.7 (0.8)	1.00	2.8 (6.2)	0.13	0.15
CG	3.4 (3.1)	4.1 (4.8)	1.00				4.3 (3.9)	0.92			

Abbreviations: EG—experimental group; CG—control group; M—Mean; MD—Mean Difference; SD—Standard Deviation; SF-12—Short Form-12 Health Survey; DASS-21—Depression, Anxiety and Stress Scale; WHIIRS—Women’s Health Initiative Insomnia Rating Scale; CVM-22—menstrual-related quality of life. Bold format indicates statistically significant results.

## Data Availability

The data presented in this study are available on request from the corresponding author. The data are not publicly available due to privacy and ethical restrictions.

## Abbreviations

The following abbreviations are used in this manuscript:

CVM-22	Menstrual-related quality of life
DASS-21	Depression, Anxiety and Stress Scale
PD	Primary Dysmenorrhea
SF-12	Short Form-12 Health Survey
WHIIRS	Women’s Health Initiative Insomnia Rating Scale
